# Power of Cognition: How Dysfunctional Cognitions and Schemas Influence Eating Behavior in Daily Life Among Individuals With Eating Disorders

**DOI:** 10.3389/fpsyg.2018.02138

**Published:** 2018-11-13

**Authors:** Tanja Legenbauer, Anne Kathrin Radix, Nick Augustat, Sabine Schütt-Strömel

**Affiliations:** ^1^LWL University Hospital Hamm for Child and Adolescent Psychiatry, Ruhr-University Bochum, Hamm, Germany; ^2^Department of Clinical Psychology and Psychotherapy, Johannes Gutenberg University Mainz, Mainz, Germany

**Keywords:** eating disorders, bulimia, binge eating, ecological momentary assessment, cognition, maladaptive schema, Young Schema Questionnaire

## Abstract

Eating disorders (EDs) are characterized by marked cognitive distortions and maladaptive schemas. Cognitive models of EDs highlight the direct impact of cognitive dysfunctions on eating-related disturbances, insofar as specific cognitive contents such as thoughts about diet rules and food or loss of control may trigger disturbed eating behavior. Moreover, early maladaptive schemas that reflect perfectionist standards and relate to achievement and performance seem to be associated with disturbed eating, e.g., via their impact on situation-specific appraisals. However, so far, no study has investigated these assumptions. Hence, the present study sought to demonstrate whether and how cognitive content exerts an impact on eating behavior in daily life, and whether maladaptive core schemas impact the occurrence of binge eating via dysfunctional ED cognitions in eating-related contexts. *N* = 29 females with bulimia nervosa (BN), *n* = 31 females with binge eating disorder (BED) and *n* = 30 female controls without EDs (NC) participated in the study. All participants received a handheld computer for a 48-h period to capture antecedents of disturbed eating behavior in daily life. Event-sampling (meals, binge eating, purging, stressful situations) and signal-sampling (five times a day) methods were applied. EMA included a short questionnaire to assess dysfunctional cognitions and level of craving and to capture information about situational contexts. Early maladaptive schemas were assessed using a short version of the Young Schema Questionnaire at baseline. The main results showed specific patterns of dysfunctional eating-related cognitions for BED and BN. Binge eating was predicted by thoughts about loss of control (positively) and dietary restraint (negatively). For meal situations, no significant differences between the two ED groups emerged. All three domains exerted indirect effects on craving via thoughts about ‘*eating/loss of control*,’ whereas neither a direct nor an indirect effect emerged regarding thoughts about ‘*dietary restraint*.’ These results fit well with previous studies and support cognitive models of EDs; schema therapeutic approaches may be a valuable contribution to enhance treatment of EDs. Further studies should explore whether the findings from emerging adulthood can be generalized to younger age groups.

## Introduction

Eating disorders (EDs) such as bulimia nervosa (BN) and binge eating disorder (BED) are common among female adolescents and emerging adults (Keski-Rahkonen and Mustelin, 2016). They are characterized by disinhibited eating behaviors, which – in the case of BN – alternate with restrictive eating episodes and purging in order to control weight. Despite differences between BN and BED regarding ED-related behaviors, the two disorders seem to share common transdiagnostic mechanisms which are involved in the maintenance of ED pathology: overvaluation of shape, weight, and eating, which are accompanied by perfectionism (Fairburn et al., 2003). It has been suggested that restrictive eating is triggered by dysfunctional thoughts (e.g., ‘I am not allowed to eat this food.’), which are associated with overvaluation of eating, weight and shape (core beliefs, e.g., ‘If I’m fat, people won’t like me.’), thus highlighting the importance of cognitive content as well as underlying dysfunctional core beliefs for the perpetuation of ED-related behaviors (Dingemans et al., 2006; Hughes et al., 2006; Unoka et al., 2010; Legenbauer et al., 2011; Oldershaw et al., 2015; Pauwels et al., 2016; Schmitz et al., 2016). Moreover, it has been hypothesized that ED-specific beliefs and thoughts indicating weight, shape and eating concerns result from more general maladaptive schemas, for instance regarding interpersonal contexts (e.g., defectiveness/shame), and consequently contribute to the development of dysfunctional ED-related symptoms and behaviors (Gongora et al., 2004; Hughes et al., 2006). However, empirical evidence is rather of a correlational nature and findings which shed light on the relationship between cognitive content and maladaptive core schemas, and how they might impact (binge) eating behavior, are lacking. Based on general cognitive models which suggest a hierarchical order of core beliefs and thoughts (e.g., Disner et al., 2011), it seems possible that core beliefs impact ED-related behaviors via situation-specific thoughts.

### Cognitive Content and Eating Behavior in EDs

Three main areas of dysfunctional cognitions have been identified in individuals with diagnosed EDs (Legenbauer et al., 2007): (a) negative thoughts about body and self-esteem, (b) thoughts about restriction of food and weight loss, and (c) thoughts about eating and loss of control. In particular, differences in the extent of thoughts about restrictive eating were found between individuals with BN and BED. By contrast, the extent of thoughts about eating and loss of control were comparable between the diagnostic categories. Moreover, the general level of dysfunctional cognitions was significantly associated with symptoms of eating disturbances. Specifically, binge eating frequency (retrospective self-report) was significantly related to the extent of thoughts about eating and loss of control, and reported levels of restrictive eating behavior were significantly linked to the extent of thoughts about dietary restraint (Legenbauer et al., 2007). There is further evidence demonstrating that change in the degree of thoughts about dietary restraint leads to change in restrained eating behavior (Legenbauer et al., 2011). In addition, some laboratory studies have indicated that craving or ED-related thoughts are elicited in specific situational contexts such as exposure to palatable food or stress induction (e.g., Stirling and Yeomans, 2004; Hilbert et al., 2007b; Siep et al., 2012; Van Dillen et al., 2013; Yokum and Stice, 2013). Finally, theoretical models hypothesize that females with disturbed eating behavior control their food intake in order to reduce weight or prevent weight gain by inhibiting thoughts about eating (palatable) food (goal conflict model; Stroebe et al., 2008). However, trying not to think about food and deliberately restraining oneself from eating results in increased rather than reduced subsequent consumption (Erskine and Georgiou, 2013). The outlined studies highlight the relevance of cognitive content and situation-specific triggers for disturbed eating behavior. However, cognitive models as well as recent research also emphasize the role of core beliefs/maladaptive schemas as triggers for dysfunctional situation-specific thoughts.

### Maladaptive Schemas

Young (1999) proposed that (early) maladaptive schemas are established in childhood, and reflect the emotional climates at that time; in particular the experience that one’s emotional needs are not satisfied. Once established, early maladaptive schemas become dysfunctional later in life because they lead to dysfunctional coping strategies that prevent needs from being met in adulthood. As such, they increase the risk of psychopathology later in life. Early maladaptive schemas are clustered into five schema domains, which in turn refer to these unmet childhood needs (e.g., Kriston et al., 2013) such as autonomy, identity, self-control or attachment. Early maladaptive schemas can be assessed using self-report questionnaires such as the Young Schema Questionnaire (YSQ; Young, 1994). Table 1 provides an overview of the various early maladaptive schemas, examples of their content, and their categorization into domains as well as associated childhood needs.

**Table 1:** Overview of schemas subsumed in schema domains and associated needs according to Young (1994, 1999).

Schema	Examples for content	Domain	Associated needs
• Failure to achieve	Inability to meet desired goals	Impaired autonomy, achievement	• Autonomy
• Dependence/incompetence	Inability to cope without support from others		• Competency
• Vulnerability to harm or illness	Inability to control the threat of disaster		• Identity
• Enmeshment/undeveloped self	Emotional over involvement with others due to fear that one will not cope without them		
• Entitlement/grandiosity	One can act without considering others	Impaired limits	• Realistic limits and self-control
• Insufficient self-control	One cannot or need not control impulses and feelings		
• Emotional deprivation • Abandonment/instability • Mistrust/abuse • Social isolation/alienation • Defectiveness/shame	Belief that one’s emotional needs will not be satisfied Danger that others will hurt/manipulate/take advantage of one Perceiving oneself as different and isolated from others Perceived defects that make one unlovable and inferior	Disconnection	• Secure attachment • Acceptance • Nurturing • Protection
• Subjugation • Self-sacrifice • Approval-seeking	Others’ desires are perceived as more important than one’s own One should focus on others’ needs, rather than one’s own	Other-directedness	• Free expression of needs and emotions
• Emotional inhibition • Unrelenting standards • Negativity/pessimism • Punitiveness	Emotional expression/experience leads to aversive consequences One should strive to achieve impossible goals	Exaggerated vigilance, inhibition	• Spontaneity and play

### The Role of Early Maladaptive Schemas in EDs

Empirical data show that - at least on a behavioral level - binge eating correlates with several early maladaptive schemas (see Table 2 for details). In particular, ‘*emotional inhibition*’ seems to be a robust predictor of binge frequency (Waller et al., 2000, 2001, 2002). Moreover, in a sample of individuals with BN, the most reliable predictor of vomiting was high ‘*defectiveness/shame*’ (Leung et al., 2000). The domain ‘*impaired autonomy/achievement*’ also appears to be of interest for binge eating in both BN and BED. In contrast, the early maladaptive schemas loading on the domain ‘*disconnection*’ as well as the early maladaptive schema ‘*self-sacrifice*’ seem to be highly relevant predominantly in BN. For individuals with BED, the domain ‘*exaggerated vigilance*,’ captured by the early maladaptive schemas ‘*unrelenting standards*’ and ‘*emotional inhibition*,’ seems to be important (Waller et al., 2003). An overview is provided in Table 2.

**Table 2:** Overview of findings indicating a relation between eating disturbances and early maladaptive schemas separated for different binge eating disorder subtypes.

Domain	Schema	Binge eating		Number of binges		Vomiting	Differentiation
		BN	BED	BN	BED	BN	BED vs. BN
Impaired autonomy, achievement	1. Failure to achieve	x ^1,2^				x ^6^	
	2. Dependence/incompetence				x ^5^		BED ↑ BN↓ x ^5^
	3. Vulnerability to harm or illness	x ^3^			x ^5^		
	4. Enmeshment/undeveloped self				x ^5^		
Impaired limits	5. Entitlement/grandiosity						
	6. Insufficient self-control	x ^1,2^					
Disconnection	7. Emotional deprivation	x ^4^					
	8. Abandonment/instability	x ^3^					BN ↑ BED↓ x ^5^
	9. Mistrust/abuse	x ^1,2^					
	10. Social isolation/alienation	x ^1,2^			x ^5^	x ^7^	
	11. Defectiveness/shame	x ^1,2^				x ^7^	
Other-directedness	12. Subjugation						
	13. Self-sacrifice						BN ↑ BED↓ x ^5^
	14. Approval-seeking						
Exaggerated vigilance, inhibition	15. Emotional inhibition						BED ↑ BN↓ x ^5^
	16. Unrelenting standards				x ^5^		
	17. Negativity/pessimism						
	18. Punitiveness						

*^*1*^Waller et al. (2001); ^*2*^Waller et al. (2001); ^*3*^Jones et al. (2005); ^*4*^Hughes et al. (2006); ^*5*^Waller et al. (2003); ^*6*^Waller et al. (2000); ^*7*^Leung et al. (2000); ^*8*^Keith et al. (2009). Note: BN = individuals with a diagnosed Bulimia nervosa, BED = individuals with a diagnosed Binge Eating Disorder.*

The outlined inconsistent findings regarding differences in the relevance of specific early maladaptive schemas and domains for BN and BED, as well as certain ED-related behaviors, might be explained by methodological issues: Most studies relied on retrospective reports to assess levels of disturbed eating behaviors, and also applied different types of assessment. Moreover, the samples varied in terms of age, and some studies included mixed diagnostic groups.

### Cognitive Antecedents of (Binge) Eating Behavior in Daily Life

To achieve a better understanding of the exact association between disturbed eating behaviors, cognitive content and maladaptive schemas, ‘ecological momentary assessment’ (EMA) techniques are helpful. EMA is an increasingly popular method to assess variables of interest in the natural environment and in real time (Stone and Shiffman, 1994; Mehl and Robbins, 2012). To our knowledge, only one EMA study has examined how cognitions influence food intake, although the study examined individuals reporting overweight and healthy-weight controls, rather than individuals with BED. Assessing eating-related cognitions immediately prior to eating events, the authors found similar levels of dysfunctional cognitions in overweight participants compared to normal-weight controls, and revealed no specific role of dysfunctional cognitions for obesity-promoting eating behavior (Boh et al., 2016). However, it remains unclear whether dysfunctional cognitions differ depending on situational contexts, e.g., meal versus binge eating or non-food-related contexts.

### Aims of the Present Study

In sum, there is evidence that specific cognitive content, namely ‘*eating and loss of control*’ as well as ‘*dietary restraint*,’ varies across diagnostic groups with binge EDs and is associated with (binge) eating behavior. Moreover, domains that reflect perfectionist standards and relate to achievement and performance as well as interpersonal insecurity and emotion regulation difficulties (e.g., ‘*impaired autonomy/achievement*,’ ‘*disconnection*,’ and ‘*exaggerated vigilance*’) also vary depending on ED category and are related to self-reported binge eating behavior. However, empirical evidence is scarce and is mostly limited to correlational or retrospective studies. Thus, it is not yet clear whether thoughts regarding loss of control contribute to binge eating, and what exact role is played by thoughts about ‘*dietary restraint.*’ Furthermore, as the association between early maladaptive schemas and cognitive content has not been investigated, the question of whether and how cognitive content and (early) maladaptive schemas interact and affect (binge) eating behavior in daily life remains unanswered.

**FIGURE 1:**
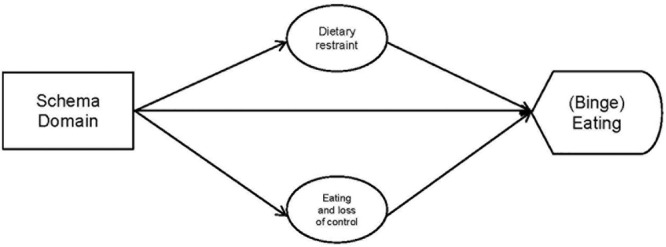
Assumed associations between schema domains, cognitive content and binge eating.

Given that beliefs/schemas tend to include rather negative/maladaptive contents, it can be assumed that they influence the interpretation of situation-specific contexts insofar as a negative (disorder-specific) appraisal is triggered (Waller et al., 2000), which in turn results in a certain (disorder-specific) behavior. Thus, it may be that the impact of schema domains on (binge) eating behavior is mediated by dysfunctional ED-specific thoughts (see Figure 1). The aim of the present study is to investigate the association of cognitive content and schema domains and their impact on (binge) eating behavior, with a special focus on a possible mediation.

The first set of research questions focuses on cognitive content and its change across different situational contexts. We assume that binge eating is associated with higher levels of thoughts about ‘*eating and loss of control*’ and, in contrast, lower levels of thoughts about ‘*dietary restraint*’ (hypothesis 1). We control for differences between individuals with a diagnosis of BN and BED in relation to cognitive content in binge and meal situations, with a special focus on change from pre- to post-binge eating/meal, as previous research indicates differences between these groups regarding cognitive content and specific eating behavior (e.g., Legenbauer et al., 2007). Finally, we assume that both the extent of thoughts relating to ‘*eating and loss of control*’ (reflecting a direct impulse for action) and ‘*dietary restraint*’ (reflecting an indirect effect of suppression/prohibition of eating) will be predictive for the occurrence of binge eating (hypothesis 2).

The second set of research questions relates to the mediation model: Based on previous research, the domains ‘*exaggerated vigilance*,’ ‘*disconnection*,’ and ‘*impaired autonomy/achievement*’ are addressed as predictors of binge eating via their impact on cognitive content. As it is not yet clear how these domains are related to ED-specific cognitive content, no specific hypotheses are formulated. Therefore, thoughts about ‘*eating and loss of control*’ as well as about ‘*dietary restraint*’ are included in the mediation analyses.

Although early maladaptive schemas are formed in childhood and adolescence, most of the aforementioned studies were performed in adult samples, when the schemas might be more pervasive and persistent (Güner, 2017). Hence, it remains unclear whether longer illness duration or developmental status interfere with the role of early maladaptive schemas in EDs. As a consequence, regarding trait early maladaptive schemas/schema domains, we control for age, as it may be the case that early maladaptive schemas exert more influence in older compared to younger individuals due to cognitive development and duration of illness. As overweight and obesity are specific conditions that might impact on early maladaptive schemas besides the ED, we additionally control for body mass index (BMI).

## Materials and Methods

### Participants

Females with a diagnosis of BN or BED volunteered to participate in the study after reading advertisements placed in local journals, across a university campus, and in an ED outpatient treatment center. The advertisements were addressed at women who experience regular binge eating episodes with or without purging behavior or restrictive eating. Simultaneously, we recruited a control group consisting of female participants who did not report any ED or other mental disorder. Reports of (subclinical) binge eating episodes or disinhibited eating led to exclusion from the NC group. In total, 90 women participated in the study, including *n* = 29 with BN (BN), *n* = 31 with BED (BED) and *n* = 30 who did not report any symptoms of EDs or other mental disorders (NC). The participants’ mean age was 28.14 years (*SD* = 7.87), with a range from 17 to 53. The mean BMI was 24.84 kg/m^2^ (*SD* = 6.59), with a range from 16.9 to 48.07 kg/m^2^. About two-thirds of the whole sample (*n* = 65) had a university entrance-level diploma and *n* = 14 had graduated from university. All others reported a lower educational level. While only one NC participant reported previous psychotherapy, *n* = 14 of the ED participants had previously attended psychotherapy, and *n* = 20 reported that they were currently receiving treatment.

### Design and Procedure

The study was conducted as part of a larger study investigating emotion regulation and cognitive processes in women with a diagnosed ED compared to healthy controls, which included an experimental task in addition to the EMA part. The results of the experimental study have not yet been published. Women who responded to the advertisements and reported binge eating episodes and restrictive eating behavior were asked during an initial telephone screening to describe their binge eating episodes to ensure that they had a clinically relevant level of impairment according to DSM-IV criteria (e.g., at least two objective binge eating episodes per week; American Psychiatric Association [APA], 1994). Those women who met this requirement and all other criteria of the screening questions of the SCID-I ED section were invited for a follow-up diagnostic interview. All others were debriefed and received advice about treatment possibilities if requested. During the interview, a clinical psychologist who was well-trained in conducting this assessment method checked for evidence of any kind of mental disorder using the German version of the SCID (Wittchen et al., 1997). All participants who met the DSM-IV criteria for BN or BED were accepted for the experimental group.

Control participants were also assessed using the screening questionnaires of the SCID-I, and all women who did not meet the criteria for any mental disorder were invited to participate. Following the diagnostic interview, participants received several questionnaires. At the start of the 48-h recording phase, all participants were given the palm (handheld) computers^1^ and were advised on how to use the device. Participants were asked to initiate an assessment if one of the following events took place: meal, binge eating, stressful situation. To assess what had been eaten during meals and binges, they filled out a food diary for each eating episode (either meal or binge). After the 48-h period, participants attended the laboratory to hand over the devices, whereupon they answered some questions referring to the handling of the EMA, and were debriefed and paid (20€).

### Measures

#### Assessment of Baseline Characteristics

The Young Schema Questionnaire – Short form (YSQ-S2; Young et al., 2003) was applied in an unpublished German version. The original questionnaire includes 95 items on 19 subscales. Since its original publication, it has been revised (YSQ-S3; Kriston et al., 2013), and one of the scales (social undesirability) has been excluded. The resulting short version (S3) therefore includes 90 items on 18 subscales. Subscale scores (range = 1–6) are calculated as the mean of the five items on each scale, with higher scores reflecting more maladaptive core beliefs. To be able to compare our findings with current research, we applied the algorithms of the S3 version to calculate scale scores and domains. For purposes of data reduction, only the early maladaptive schemas and schema domains that qualified based on previous research (those that had been associated with binge eating in BN and BED; e.g., Waller et al., 2000) were included in the analyses (domains: ‘*exaggerated vigilance*,’ ‘*disconnection*,’ and ‘*exaggerated inhibition*’; single early maladaptive schemas: ‘*unrelenting standards*’ and ‘*emotional inhibition*’). Subscales and domains according to the S3 version (Kriston et al., 2013) are displayed in Table 1. The S3 version proved to be reliable, with acceptable factorial validity. Internal consistency was found to be sufficient (Cronbach’s α > 0.70), with the exception of the scale ‘*Entitlement/Grandiosity*’ (Cronbach’s 0.67). Schema scores were positively associated with measures of psychopathology, and based on schema scores; it was possible to differentiate subgroups with different levels of healthcare utilization.

The Eating Disorder Examination Questionnaire (EDE-Q; Fairburn and Beglin, 1994) assesses the degree of eating-disordered psychopathological behavior in the past 28 days according to the following four dimensions: ‘restraint eating,’ ‘eating concerns,’ ‘weight concerns,’ and ‘shape concerns.’ The German version of the EDE-Q (Hilbert et al., 2007a) shows good convergent and discriminatory validity, as well as high reliability and test–retest reliability (Hilbert et al., 2012).

The Beck Depression Inventory-II (BDI-II), a 21-item self-rating scale, was administered to assess depressive symptoms. The inventory assesses the severity of depressive symptoms using a sum score; the clinical cut-off score is 20. The German version has shown good internal consistency (Cronbach’s α = 0.73–0.92; Beck et al., 1988) and good test–retest reliability (*r*_tt_ = 0.75) in a non-clinical population (Besier et al., 2008).

#### Ecological Momentary Assessment of Cognitions

For data acquisition regarding cognitions and eating behavior, we used an EMA approach with a signal-sampling (five times a day) and event-sampling design. For the event sampling, participants were instructed to answer electronic questionnaires before and after a meal or binge eating took place. Healthy control women received the same instructions, but it was noted that binge eating may not be of relevance for them. With regard to signal sampling, an acoustic signal reminded the participant to start an assessment. The 48-h period began in the afternoon on day 1, the first assessment took place between 3 pm and 4 pm, and the second fixed assessment was scheduled between 7 pm and 8 pm. On the second day, four fixed assessments were planned: between 10 and 11 am, 1 and 2 pm, 4 and 5 pm, and 8 and 9 pm. On the third day, two more assessments were programmed between 9 and 10 am and at lunchtime (between noon and 1 pm), before the handheld device was returned. For all assessments, the questions referred to (a) persons present when the event took place (e.g., friends, family, strangers), (b) location (at home, work, etc.), and (c) the type of situation. With regard to the type of situation, the main categories were meal (specification pre or post), binge eating (specification pre or post), purging behaviors (specification pre or post), and ‘stressful experiences.’ There was also the possibility to classify situations that did not fit into these categories as ‘neutral’ or ‘non-neutral.’ However, to capture more specifically which situations were classified as ‘neutral,’ ‘non-neutral,’ or ‘stressful experiences,’ participants had to further categorize these situations as (1) eating-related context, (2) body-related context, (3) achievement-related context, or (4) interpersonal. Next, (d) the presence of dysfunctional cognitions was assessed using a state version of the EDCQ (27 items relating to cognitive content of the last 5 min; for further details on the instrument see below). To capture (e) mood state/emotions, a shortened version of the PANAS, with five items on each scale, was incorporated in the electronic questionnaire. In addition, participants could choose one of the following options (1) ‘I feel something, but it is not clear/easy to identify,’ (2) ‘I feel something, but I can’t describe it properly,’ (3) ‘I feel something, but I can’t deal with it,’ and (4) ‘I feel nothing.’ Finally, participants had to judge how strong their craving for food/binge eating was (scale 0 to 10) and to what extent they were refraining from eating (scale 0 to 10). All questions during the assessment referred to the last 5 min.

The German-language ‘Eating Disorder Cognition Questionnaire’ (EDCQ; Legenbauer et al., 2007) is an instrument used to assess cognitions related to ED symptoms. The items evaluate the frequency of cognitions during the previous week and are answered on a four-point Likert scale (0 = not at all, 3 = always). It consists of three subscales (‘*body and self-esteem*,’ ‘*dietary restraint*,’ and ‘*eating/loss of control*’). As only the eating-related scales were of interest for the present study, only these are described here: The subscale ‘*dietary restraint*’ (DR) includes nine items (range 0–27) assessing thoughts about restrained eating behavior and weight loss, for example: ‘Tomorrow I will fast’ and ‘I have to resist and may eat nothing.’ The subscale ‘*eating and loss of control*’ (eight items, range 0–24) includes thoughts about binge eating behavior, craving, and loss of control (e.g., ‘I will lose control immediately and have a binge’; ‘I feel a craving to eat’). The subscales have shown good internal consistency (for further details, see Legenbauer et al., 2007). The scale has been applied as state and trait versions in several experimental and treatment studies in ED samples (e.g., Legenbauer et al., 2008, 2011; Rühl et al., 2011).

#### Hardware and Software

For the EMA, the Palm HP iPAQ rx1950^i^, a handheld computer, was used. Participants responded to the items using a stylus and a touchscreen. The electronic questionnaires were programmed in an early version of the ‘psychoEQ’^®^ assessment software^2^.

### Ethics Statement

The study was approved by the ethics committee of the German Society for Psychology. The study protocol was conducted in accordance with the Declaration of Helsinki (revised 1983). Written informed consent was provided by all participants, who were aware that they could withdraw from the experiment at any time without further consequences.

### Data Analyses

Statistical analyses were performed using IBM^®^ SPSS^®^ Statistics version 24, and in the case of ‘generalized linear mixed models,’ and ‘Wilcoxon signed-rank test,’ the software ‘R,’ version 3.5.1^®^ The R foundation. To check for differences at baseline between the three groups (BN, BED, NC), Chi^2^ tests and (M)ANOVAs were performed for psychosocial and disorder-related data as well as for relevant questionnaires (YSQ subscales and domains, BDI). As BMI differed between the groups, it was considered as covariate in the (M)ANOVA analyses in order to control for its influence on cognition, early maladaptive schemas/domains and eating behavior. The results of the (M)ANCOVAs are only reported when the covariate showed a significant influence.

To test for differences in cognitive content depending on situational contexts, generalized linear mixed models were performed with case number as random effect, number of events as repeated measure and situation specifics as fixed effects. In addition, to explore differences between the ED subtypes with regard to cognitive content in eating-related contexts, conservative Wilcoxon rank sum tests with continuity correction were performed, assessing changes in cognitive content pre-to post-binge eating and pre- to post-meal, separately for BN and BED. False discovery rate (FDR) correction to adjust for multiple testing was applied where necessary. Results of FDR correction were reported when relevant. Finally, a (mixed model) logistic regression with each single participant as random effect, both EDCQ scales as fixed effects, and the occurrence of binge eating as dependent variable, was performed to estimate the predictive value of both thoughts about ‘*dietary restraint*’ and ‘*eating and loss of control*’ for the occurrence of binge eating. Diagnostic group was included as covariate. To capture the influence of early maladaptive schemas on dysfunctional cognition, and how these affect eating behavior in a natural environment, three regression analyses were performed using a macro for multilevel mediation (MLmed) provided by Rockwood and Hayes (2017). As the macro does not allow the inclusion of a dichotomous variable as dependent variable, and the number of binge eating episodes was rather low and thus limited the validity of the results, the occurrence of binge eating was operationalized as the level of craving, which is strongly related to binge eating and disinhibited food intake (e.g., Van den Akker et al., 2018). Level of craving therefore served as dependent variable (y). The level-two variable within the multilevel dataset was defined as case number. Additionally, diagnostic status was included as a level-two covariate. The two EDCQ subscales (‘*eating and loss of control*,’ ‘*dietary restraint*’) were incorporated into the model as mediators M1 and M2. To detect possible indirect effects, the robust bootstrapping method was applied, which relies on confidence intervals (e.g., Hayes and Scharkow, 2013). The macro applies bootstrapping with 10,000 replicates. The analyses with MLmed relied on data from pre-binge situations and neutral situations, as the difference in craving levels were the most marked between these types of situations^3^.

## Results

### Baseline Differences

There were no differences between the groups with regard to age. Moreover, the groups contained equal proportions of participants classified as emerging adults [χ^2^(2, 90) = 1.503, *p* = 0.472]. In total *n* = 57 of the total sample, participants (*n* = 90) were categorized as ‘emerging adults’ (BN = 20, BED = 17, NC = 20). In contrast, BMI was higher in BED compared to BN (*p* = 0.018) and NC (*p* < 0.001). The educational level was comparable between the groups [χ^2^(2, 90) = 10.075, *p* = 0.260].

Eating pathology assessed using the EDE-Q was markedly higher in the two ED groups compared to NC on all scales. BMI as covariate showed a statistically significant influence on the three ‘concern’ scales of the EDE-Q [eating concern: *F*(1,85) = 5.583, *p* = 0.021; weight concern *F*(1,85) = 7.646, *p* = 0.007; shape concern *F*(1,85) = 5.305, *p* = 0.024]. *Post hoc* analyses revealed a comparable level of weight concern and shape concern between BN and BED, whereas BN reported higher levels of restraint eating (*p* < 0.001) and eating concern (*p* = 0.012) compared to BED. Regarding comorbidity, both ED groups (*p* < 0.001) reported clinically relevant levels of depression (BDI) compared to NC (*p* < 0.001). No statistically significant *post hoc* differences emerged between BED and BN; however, from a clinical perspective, BN exceeded the cut-off criteria for clinically relevant depression (BDI-II > 20), whereas the BED participants reported moderate levels of depression on average (for details see Table 3).

**Table 3:** Means and standard deviations of sample characteristics and relevant early maladaptive schema (EMS) and schema domains.

	BN (*N* = 29)		BED (*N* = 30)		NC (*N* = 30)		Statistics		
	*M*	*SD*	*M*	*SD*	*M*	*SD*	*F*	*p*	*Post hoc*
Age (in years)	27.00	8.41	30.48	8.50	26.83	6.17	2.149	0.123	F-BN = F-BED = F-NC
BMI (in kg/m^2^)	24.30	7.01	28.61	7.21	21.45	2.11	11.252	<0.001	F-BED > F-BN = F-NC
BDI-II	22.33	10.58	19.74	9.04	2.90	2.76	49.751	<0.001	F-BN = F-BED > F-NC
**EDE-Q**									
Restraint eating	3.93	1.40	2.42	1.90	0.39	0.57	61.984	<0.001	F-BN > F-BED > F-NC
Eating concern	3.55	1.50	2.61	1.45	0.10	0.13	61.905	<0.001	F-BN > F-BED > F-NC
Weight concern	4.60	1.43	4.41	1.20	0.76	0.89	95.038	<0.001	F-BN = F-BED > F-NC
Shape concern	4.26	1.48	3.79	1.28	0.54	0.83	78.497	<0.001	F-BN = F-BED > F-NC
**Subscales YSQ**									
Emotional inhibition	2.79	1.19	2.86	1.05	1.45	0.61	19.650	<0.001	F-BN = F-BED > F-NC
Unrelenting standards	4.67	1.09	4.50	0.87	2.47	1.06	30.318	<0.001	F-BN = F-BED > F-NC
**Schema domains**									
Impaired autonomy, achievement^∗^	3.67	0.99	3.43	1.00	1.62	0.58	48.634	<0.001	F-BN = F-BED > F-NC
Disconnection^∗^	3.78	0.91	3.23	0.67	1.99	0.69	27.380	<0.001	F-BN = F-BED > F-NC
Exaggerated vigilance	3.69	0.95	3.52	0.68	1.97	0.53	48.847	<0.001	F-BN = F-BED > F-NC

*M, mean; *SD*, standard deviation; *N*, number; *BN*, females with a diagnosis of bulimia nervosa; *BED*, females with a diagnosis of binge eating disorder; *NC*, females without a diagnosis of eating disorders; *BMI*, body mass index (kg/m^*2*^); *BDI*, Beck Depression Inventory; *YSQ-S2*, Young Schema Questionnaire – Short version. ^∗^ BMI as covariate exerted a significant influence on the following domains: Disconnection *F*(1,89) = 4.454, *p* = 0.038; impaired autonomy *F*(1,89) = 8.055, *p* = 0.006.*

With regard to early maladaptive schemas, women of both ED groups showed strongly elevated scores on both included YSQ subscales compared to NC. There were no differences between BN and BED. Similar results emerged when schema domains were compared between the groups: There was no significant difference between BN and BED, whereas both ED groups showed significantly higher scores compared to NC on the three included domains. With the exception of the domain ‘*exaggerated vigilance/inhibition*,’ BMI as covariate showed a statistically significant difference. Details of the MAN(C)OVA and covariate statistics are presented in Table 3.

### Variations in Cognitive Content Across Situational Contexts

In total, 864 events were recorded over all participants. Of these, *n* = 382 were categorized as neutral situations (44.2%), *n* = 101 as subjectively stressful experiences (11.7%), *n* = 296 events were meal-related, of which *n* = 144 were pre-meal (16.7%) and *n* = 152 post-meal (17.6%), *n* = 57 were binge-related, with *n* = 20 entries prior to a binge (*n* = 20, 2.3%) and *n* = 37 entries post-binge (4.3%). Additionally, a small number of entries related to purging behavior [*n* = 11 (1.3%) before purging took place and *n* = 17 (2.0%) after purging was captured].

#### Results Relating to Hypothesis 1

**FIGURE 2:**
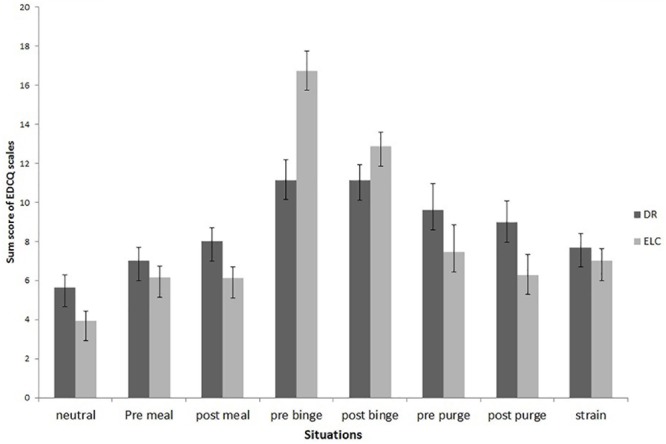
Change of cognitive content depending on situational context. DR, thoughts about dietary restraint; ELC, thoughts about eating and loss of control; pre meal, pre meal situations; post meal, post meal situations; pre binge, pre binge situations; post binge, post binge situation; pre purge, situation before compensatory behavior took place; post purge, situation after compensatory behavior; strain, situation subjectively experienced as stressful. Eating Disorder Cognition Questionnaire (EDCQ).

The extent of thoughts about ‘*dietary restraint*’ differed significantly depending on the type of situation [*F*(7,318) = 21.971, *p* < 0.001]. The highest extent was reported in binge eating situations compared to neutral situations (*p* < 0.001). Moreover, the extent of thoughts about ‘*eating and loss of control*’ also differed significantly depending on the type of situation [*F*(7,239) = 51.125, *p* < 0.001]. The highest extent was reported in binge eating associated contexts before a binge took place compared to neutral situations (*p* < 0.001). The change in the extent of thoughts about both ‘*dietary restraint*’ and ‘*eating and loss of control*’ depending on the type of situation is displayed in Figure 2.

*Post hoc* Wilcoxon rank sum tests focused on differences in cognitive content from pre- to post-binge eating, taking into account the type of ED. For BN, changes in thoughts associated with ‘*dietary restraint*’ were marginally significant, indicating a slight increase from pre- (*M* = 11.15, *SE* = 9.32) to post-binge (*M* = 14.86, *SE* = 13.38; *W* = 99.5, *p* = 0.091, Cohen’s *d* = -0.055), whereas no significant change emerged for individuals with BED (pre: *M* = 12.0, *SE* = 9.76, post: *M* = 11.07, *SE* = 9.80; *W* = 39.5, *p* = 0.354, Cohen’s *d* = 0.20). Regarding meal situations, no significant change emerged, either for individuals with BN (pre: *M* = 11.93, *SE* = 10.66, post: *M* = 12.71, *SE* = 11.66; *W* = 932.5, *p* = 0.666, Cohen’s *d* = -0.10) or for those with BED (pre: *M* = 8.04, *SE* = 7.13, post: *M* = 10.47, *SE* = 9.49; *W* = 996.5, *p* = 0.198, Cohen’s *d* = 0.20).

With respect to cognitions relating to ‘*eating and loss of control*,’ *post hoc* Wilcoxon sum rank tests also revealed no significant change from pre- (*M* = 15.92, *SE* = 13.79) to post-binge eating episode (*M* = 14.62, *SE* = 13.26) in individuals with BN (*W* = 152.5, *p* = 0.291, Cohen’s *d* = 0.20), whereas in individuals with BED, the level of cognitions decreased marginally significantly from pre- (*M* = 15.92, *SE* = 13.79) to post-binge eating episode (*M* = 10.47, *SE* = 9.49; *W* = 53, *p* = 0.052, Cohen’s *d* = 1.04). Regarding meal situations, no significant change emerged, either for individuals with BN (pre: *M* = 9.49, *SE* = 8.38, post: *M* = 8.94, *SE* = 7.95; *W* = 1039, *p* = 0.327, Cohen’s *d* = 0.08) or for those with BED (pre: *M* = 7.89, *SE* = 7.15, post: *M* = 7.47, *SE* = 6.44; *W* = 929, *p* = 0.401, Cohen’s *d* = 0.08).

### Impact of Cognitive Content on (Binge) Eating Behavior

#### Results Regarding Hypothesis 2

The logistic regression showed that both scales were of significant predictive value for binge eating: ‘*eating and loss of control*’ exerted a positive effect on binge eating occurrence (β = 0.352, *p* < 0.001) and ‘*dietary restraint*’ was negatively related to binge eating (β = -0.092, *p* = 0.041). Diagnostic group as covariate was not significant (*p* = 0.208).

### Impact of Early Maladaptive Schemas and Schema Domains on Cognitive Content in (Binge) Eating Situations

#### Results Regarding the Second Set of Research Questions

The first mediation model regressed the domain ‘*impaired autonomy/achievement*’ on craving intensity. The two cognitions about ‘*dietary restraint*’ and ‘*eating and loss of control*’ were included as possible mediators. A full mediation model emerged for the pathway *eating and loss of control*,’ with a statistically significant indirect effect (*Z* = 2.976; *p* = 0.003; β = 0.564, CI [0.233, 0.975]) and a non-significant direct effect of the domain on level of craving (β = -0.305, CI [-0.738, 0.126]). No mediation effect emerged for the ‘*dietary restraint*’ pathway (β = -0.207, CI [-0.535, 0.061]. However, the extent of ‘*impaired autonomy/achievement*’ directly impacted the extent of thoughts about ‘*dietary restraint*’ (β = 2.480; *t* = 4.506, *p* < 0.001, CI [1.385, 3.575]) and about ‘*eating and loss of control*’ (β = 1.764; *t* = 4.339, *p* < 0.001, CI [0.950, 2.572]). Moreover, the pathway from ‘*eating and loss of control*’ to craving was statistically significant (β = 0.320; *t* = 4.196, *p* < 0.001, CI [0.168, 0.472]), whereas the pathway from thoughts about ‘*dietary restraint*’ to craving was not significant (β = -0.083, CI [-0.194, 0.028]). The diagnostic category only had an impact on the ‘*eating and loss of control*’ pathway (*p* < 0.050).

Regarding the impact of ‘*disconnection*’ on binge eating behavior, again, an indirect effect of schema domain via ‘*eating and loss of control*’ on level of craving emerged (*Z* = 2.170; *p* = 0.030; β = 0.434, CI [0.103, 0.869]), but there was no direct effect of ‘*disconnection*’ (β = -0.204, CI [-0.692, 0.283]), showing a full mediation effect: Higher scores in the domain ‘*disconnection*’ were associated with greater thoughts about ‘*eating and loss of control*,’ which in turn led to a higher level of craving. The schema domain directly impacted the extent of thoughts about ‘*dietary restraint*’ (β = 2.263; *t* = 3.117, *p* = 0.003, CI [0.818, 3.707]) and also about ‘*eating and loss of control*’ (β = 1.409; *t* = 2.645, *p* = 0.010, CI [0.348, 2.471]), but there was no significant pathway from ‘*dietary restraint*’ to craving (β = -0.092, CI [-0.202, 0.019]). The diagnostic category reached significance as a covariate for the ‘*eating and loss of control*’ pathway (*p* < 0.050) and the pathway from domain to cognition about ‘*dietary restraint*’ (*p* < 0.010).

**FIGURE 3:**
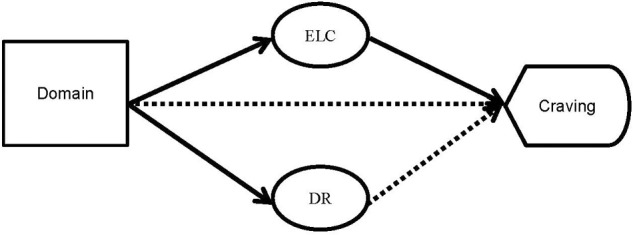
Mediation model for the overall pattern of associations between domains, cognitive content and craving. *N* = 377 data sets from neutral as well as pre-binge EMA assessments were included from all available participants. Domains refer to either ‘*impaired autonomy/achievement*,’ ‘*disconnection*,’ or ‘*exaggerated inhibition.*’ DR, thoughts about dietary restraint; ELC, thoughts about eating and loss of control; **—-** statistically significant pathway, **■ ■ ■ ■** non-significant pathway.

The final model assessed the impact of the domain ‘*exaggerated vigilance*’ on craving. Again, an indirect effect of schema domain emerged, indicating a mediation by thoughts about ‘*eating and loss of control*’ (*Z* = 2.437; *p* = 0.015; β = 0.468; CI [0.146, 0.898]). No direct effect of ‘*exaggerated vigilance*’ (β = -0.249, CI [-0.745, 0.247]) emerged, showing a full mediation effect: Higher scores in the domain ‘*exaggerated vigilance*’ were associated with greater thoughts about ‘*eating and loss of control*,’ which in turn led to a greater craving. Again, the schema domain directly impacted the extent of thoughts about ‘*dietary restraint*’ (β = 2.741; *t* = 4.308, *p* ≤ 0.001, CI [1.475, 4.007]) and about ‘*eating and loss of control*’ (β = 1.549; *t* = 3.169, *p* = 0.002, CI [0.576, 2.521]); diagnostic category as a covariate reached significance for both ‘*eating and loss of control*’ pathways (*p* < 0.001). Because the pathways were robust across the different domains, the mediation model is summarized within Figure 3.

## Discussion

The main aim of the present study was to demonstrate how cognitive content, maladaptive schemas and eating behavior in daily life are related, with a specific focus on the mediating role of cognitive content between early maladaptive schemas/schema domains and (binge) eating behavior in daily life. To achieve this, an EMA approach was chosen using event- and signal-sampling techniques to capture eating behavior in daily life over a 48 h-period as well as associated levels of dysfunctional cognitions. Schema domains were assessed at baseline. The results showed specific patterns of eating-related cognitions for BED and BN. In BED, ‘*eating and loss of control*’ was marginally significant reduced from pre- to post-binge eating episode, whereas no statistically significant reduction emerged in BN. In contrast, BN showed a trend toward an increase in thoughts about ‘*dietary restraint*’ from pre to post binge. Moreover, binge eating was positively predicted by thoughts about ‘*eating and loss of control*’ as well as negatively by thoughts about ‘*dietary restraint.*’ With regard to meal situations, no significant differences between the two ED groups emerged. In terms of the influence of schemas on eating behavior, high levels on schema domains led to greater thoughts about ‘*eating and loss of control*’ as well as ‘*dietary restraint.*’ No direct effect on craving was found, whereas a robust pattern emerged indicating an indirect effect of schema domains via thoughts about ‘*eating and loss of control*’ on the level of craving. By contrast, thoughts about ‘*dietary restraint*’ did not impact levels of craving. These results support previous research and reflect core assumptions of cognitive models of EDs (e.g., Thompson et al., 1999; Fairburn et al., 2003; Farrell et al., 2006).

### Relevance of Thoughts for Eating Behavior

Despite the often postulated relevance of cognitions for eating behavior, empirical evidence that includes ecological data on the association between cognition and eating is still scarce (Boh et al., 2016). Similar to the study by Boh et al. (2016), the present study failed to show a significant impact of (eating-related) dysfunctional cognitions in normal meal situations. However, with regard to binge eating situations, our findings highlight the impact of cognitive content on binge eating behavior. This discrepancy may be due to methodological issues (e.g., free entry format that was subsumed in broader, possibly unspecific categories vs. validated, ED-related questionnaire; obese sample vs. clinical sample with higher ED-related psychopathology). However, contrary to our first hypothesis that thoughts about ‘*eating and loss of control*’ would change from pre- to post-binge eating in both ED groups, this was only true for the group of individuals with BED, and not for those with BN. It may be that the underlying processes that trigger craving and binge eating are more complex in BN compared to BED, thus leading to slower decreases in these cognitions, which were not captured within the time frame assessed in the present study. Moreover, due to the possibility to compensate for binge eating through purging, individuals with BN might not experience a significant reduction in thoughts about ‘*eating and loss of control*,’ as they are always able to continue the binge. On the other hand, cognitions relating to ‘*dietary restraint*’ increased after binge eating in BN, which might reflect the fear of weight gain and might both trigger and maintain the alternations between binging and dieting.

We assumed that a reduction in ‘*dietary restraint*’ and at the same time an increase in thoughts about ‘*eating and loss of control*’ would predict binge eating, but both contents were highest before binge eating. This may reflect a highly psychopathological or aroused state prior to binge eating, leading to high levels of eating-related dysfunctional cognitions. It may also indicate the internal struggle between the craving and the wish to lose weight (conflicting goals, Stroebe et al., 2008). Both points fit well into Heatherton and Baumeister’s (1991) model of binging as an escape from negative self-awareness, and are in line with research showing that increases in negative affect precede binge eating, whereas positive affect decreases prior to binge episodes (e.g., Smyth et al., 2007; Berg et al., 2013; Wonderlich et al., 2018). However, due to the small number of binge events and the small period of time assessed in the present study, it was not possible to conduct more sophisticated analyses on the course of cognitions within 1 h prior to binge eating. In sum, the present results support the assumptions of cognitive models on EDs which highlight the impact of dysfunctional cognitions on eating-related behavior.

### Role of Schema Domains for Binge Eating in Daily Life

This is the first study to address the question of whether schema domains impact on binge eating in a natural environment by eliciting dysfunctional cognitions. We focused on domains that clustered together single early maladaptive schemas relating to achievement and performance, as well as interpersonal problems. These reflect the perfectionist thinking of individuals with BN (Esposito et al., 2017; Levinson et al., 2017) and may therefore be of overall importance for eliciting dysfunctional thoughts about ‘*dietary restraint*’ or ‘*eating and loss of control*’ in individuals with EDs.

To test the assumed mediation model, we relied on craving as a marker for binge eating, as previous research suggests that craving precedes binge eating (e.g., Legenbauer et al., 2004; Coelho et al., 2014; Van den Akker et al., 2018). The results imply a robust pattern of mediation for all three domains on craving via thoughts about ‘*eating and loss of control*,’ but not via thoughts about ‘*dietary restraint.*’ It may be that the pathway indicates dysfunctional emotion regulation by binge eating or purging caused by these schema domains. It is possible that cognitions relating to ‘*eating and loss of control*’ emerge as a consequence of negative affect, as predicted by the escape model. The ‘*eating and loss of control*’ scale reflects disinhibition in eating behavior, thoughts about when and what can be eaten, as well as craving. Therefore, it may reflect facets of impulsivity, which has recently been associated with binge eating. For example, the association between negative affect and binge eating was found to be strengthened by higher levels of non-planning impulsivity (Mason et al., 2018). Other facets of impulsivity, such as reward sensitivity or delay discounting, may also trigger thoughts about ‘*eating and loss of control*.’

Moreover, all three domains include maladaptive schemas which reflect perfectionism and high achievement orientation (e.g., domain ‘*impaired autonomy*’) as well as interpersonal issues (e.g., domain ‘*disconnection*,’ Tezel et al., 2015) and negative self-related thinking (e.g., Thew et al., 2017). It may be that high scores in these domains lead to negative appraisals in interpersonal as well as performance-related contexts. This idea is supported by the interpersonal model of binge eating, and complements recent evidence from a study using EMA technology, which demonstrated an impact of negative affect on binge eating, mediated by interpersonal problems (Ambwani et al., 2015). In particular, the authors showed that the presence of interpersonal problems in general intensified the association between momentary interpersonal perceptions and binge eating behavior. Furthermore, in women with BN, worse-than-average social interactions pre-binge and a deterioration in perceived social interactions post-binge have been described (Steiger et al., 1999). Possibly, interpersonal problems, e.g., agentic rather than communal or inflexible behaviors, shape experiences of negative affect, thus leading to rather negative appraisals of momentary interpersonal perceptions of social interactions; and early maladaptive schemas and schema domains might play a crucial role in these processes. Thus, future research is needed to further explore the postulated processes.

In sum, by using domains instead of single early maladaptive schemas to predict the impact on binge eating, it was possible to overcome difficulties of previous studies. Furthermore, we found strong evidence for the association of cognitive content in specific situational contexts in daily life with trait maladaptive schemas and with domains. Thus, our findings support assumptions from cognitive models (Thompson et al., 1999; Fairburn et al., 2003; Farrell et al., 2006) and may contribute to a further adaptation of intervention strategies. For instance, there is first evidence indicating that schemas change during treatment and that certain schemas may facilitate or inhibit improvements (Cullum, 2009; Pugh, 2015; McIntosh et al., 2016).

Finally, it should be taken into account that some of the previous studies included mixed samples of binge eating EDs, and only a small number of studies assessed schema domains. For example, the sample of Waller et al. (2000) also included individuals with anorexia nervosa purging type, and the sample was younger, with a lower age range than that of the present study. No significant influence of age was detected: Younger participants classified as emerging adults exhibited comparable patterns of early maladaptive schemas to those of older participants. Early maladaptive schemas are formed in childhood and adolescence, with some schemas emerging later than others (e.g., self-sacrifice, unrelenting standards and emotional inhibition develop in later stages; Güner, 2017). As we did not find any age-related differences, it appears that early maladaptive schemas were already stable in the group of emerging adults. Future research should therefore focus on adolescents with binge eating EDs in order to capture developmental changes in early maladaptive schemas.

### Limitations

The strength of the present study lies in the assessment of cognitive content in relation to binge eating with the use of an innovative technology in a sample of women with two subtypes of clinically diagnosed EDs and a healthy control group. However, several limitations should be addressed: (1) The duration of 48 h is very short compared to other EMA studies. This limited the number of events, in particular binge eating episodes, and purging behavior was also less frequent. We therefore used craving as dependent variable in our mediation models, potentially impacting the outcome, as no direct comparison of binge vs. non-binge situations was performed. However, the present study was planned as a pre-study for a grant application to test the feasibility of this approach (assessing cognitive content with a questionnaire within real-life contexts; each assessment therefore took about 10 min) and to provide first data on cognitive content in binge eating episodes. The duration was therefore limited to 48 h in order to limit the burden on the participants and to reduce dropout due to missing data or ‘work overload’ with the EMA documentation. (2) The reported data correspond to situational contexts in which a binge is about to start, and the intensity of thoughts about ‘*eating and loss of control*’ may be tempered by the binge process. However, after a binge took place, participants answered the questions again, and the level of thoughts about ‘*eating and loss of control*’ then decreased, underlining the relevance of these cognitions before the binge took place. Nevertheless, it has to be considered that as the process of binge eating had already begun when participants answered the questionnaires, high levels of thoughts about ‘*eating and loss of control*’ might not initially have triggered the process, but might have increased due to the process. Hence, we cannot draw conclusions about the causality regarding the relationship between cognition and binge eating. It may be a bidirectional pathway. (3) As early maladaptive schemas were not assessed during the EMA, we may have missed important information regarding the influence of early maladaptive schemas on cognitive processes due to the exclusion of the YSQ from the EMA. Nevertheless, it was important to keep the number of items at each assessment time point to a minimum in order to maintain motivation for data entries over the sampling period. (4) We regressed early maladaptive schemas/schema domains on craving with only two types of ED-specific thoughts as mediators, which limits the generalizability of the data. (5) It appeared that binge eating episodes and meals were not recorded at all times, because some participants had only one or two meals in their record over the 48 h assessment. Therefore, it may be that the data regarding the eating-related contexts were selective, meaning that the results should be interpreted with caution. (6) We did not include information about the type and amount of food, although this might also influence the level of dysfunctional cognition. For instance, breaking dietary rules might have led to higher negative affect or marked thoughts about loss of control. (7) We did not take into account purging behavior, which might further account for differences between the two ED subtypes, because the number of events was too small for analyses. (8) The duration of EMA might have been too short to capture variations in eating behavior. Therefore, replication of the findings is needed, as the limited number of binge eating episodes reduces the generalizability of the data. (9) Finally, the sample is rather small, and more sophisticated analyses of EMA data, for example using structural equation modeling, would require more participants and events.

## Conclusion

To conclude, empirical evidence regarding the role of cognitive content and early maladaptive schemas in various aspects of eating pathology, namely disturbed eating behavior and pathological attitudes, appears to be robust. Nevertheless, existing evidence is limited due to the correlational nature of the data and the focus on trait aspects. Moreover, the role of schema domains has been neglected so far (Pugh, 2015). The present study therefore adds to the understanding of how early maladaptive schemas and schema domains might exert their effect on eating behavior. Despite the incoherent picture, empirical evidence emerged for the impact of single domains on situation-specific cognitions and their behavioral consequences such as restricted or disinhibited eating.

Future studies could integrate assessments of schema and coping modes, as these have been linked to disordered eating behaviors. For instance, it has been postulated that binge eating reduces affect after schema activation in terms of secondary avoidance; adaptive coping modes facilitate the avoidance of affect associated with schema activation (Simpson, 2012). Based on this evidence, schema therapy methods may be a promising addition to cognitive-behavioral treatments for binge eating EDs.

## Author Contributions

TL and SS-S designed the study. SS-S conducted the study. TL and SS-S conducted the literature review and wrote the research summaries. All authors planned the analyses and TL and NA analyzed the data. TL and SS-S wrote the first draft of the manuscript. All authors contributed to and have approved the final manuscript. All authors had full access to the study data.

## Conflict of Interest Statement

The authors declare that the research was conducted in the absence of any commercial or financial relationships that could be construed as a potential conflict of interest.

## Footnotes

1 The study was conducted between 2006 and 2008. At that time, the palm handheld was the most sophisticated device for an EMA study.

2 https://www.psychoware.de/psychoeq/

3 Mean score of craving for binge situations *M* = 8.49 (*SE* = 0.66; CI [7.2, 9.8]), for neutral situations *M* = 3.35 (*SE* = 0.14, CI [3.1, 3.6]), *F*(242,1) = 57.96, *p* < 0.001).
